# Characterizing dynamic regulatory programs in mouse lung development and their potential association with tumourigenesis via miRNA-TF-mRNA circuits

**DOI:** 10.1186/1752-0509-7-S2-S11

**Published:** 2013-12-17

**Authors:** Juan Liu, Xinghuo Ye, Fang-Xiang Wu

**Affiliations:** 1 School of Computer, Wuhan University, Wuhan 430072, China; 2 Division of Biomedical Engineering, University of Saskatchewan, Saskatoon, SK S7N 5A9, Canada

## Abstract

**Background:**

In dynamic biological processes, genes, transcription factors(TF) and microRNAs(miRNAs) play vital regulation roles. Many researchers have focused on the transcription factors or miRNAs in transcriptional or post transcriptional stage, respectively. However, the transcriptional regulation and post transcriptional regulation is not isolated in the whole dynamic biological processes, there are few reserchers who have tried to consider the network composed by genes, miRNAs and TFs in this dynamic biological processes, especially in the mouse lung development. Moreover, it is widely acknowledged that cancer is a kind of developmental disorders, and some of pathways involved in tissue development might be also implicated in causing cancer. Although it has been found that many genes differentially expressed during mouse lung development are also differentially expressed in lung cancer, very little work has been reported to elucidate the combinational regulatory programs of such kind of associations.

**Results:**

In order to investigate the association of transcriptional and post-transcriptional regulating activities in the mouse lung development, we define the significant triple relations among miRNAs, TFs and mRNAs as circuits. From the lung development time course data GSE21053, we mine 142610 circuit candidates including 96 TFs, 129 miRNAs and 13403 genes. After removing genes with little variation along different time points, we finally find 64760 circuit candidates, containing 8299 genes, 50 TFs, and 118 miRNAs in total. Further analysis on the circuits shows that the circuits vary in different stages of the lung development and play different roles. By investigating the circuits in the context of lung specific genes, we identify out the regulatory combinations for lung specific genes, as well as for those lung non-specific genes. Moreover, we show that the lung non-specific genes involved circuits are functionally related to the lung development. Noticing that some tissue developmental systems may be involved in tumourigenesis, we also check the cancer genes involved circuits, trying to find out their regulatory program, which would be useful for the research of lung cancer.

**Conclusions:**

The relevant transcriptional or post-transcriptional factors and their roles involved in the mouse lung development are both changed greatly in different stages. By investigating the cancer genes involved circuits, we can find miRNAs/TFs playing important roles in tumour progression. Therefore, the miRNA-TF-mRNA circuits can be used in wide translational biomedicine studies, and can provide potential drug targets towards the treatment of lung cancer.

## Background

Although it is not completely understood what and how many factors play main roles in the process of lung development, several factors that invovle in the dynamic process and play important roles have been confirmed by the biologist. Through analysis of gene expression data at different stages of lung development in the field of molecular biology, a series of novel temporal regulations, target genes, transcription factors, and candidate regulatory pathways have been observed during these dynamic biological processions [1,2]. The mammalian lung development can be divided into six stages, ranging from embryos to mature lung [1,3]. At these different stages of lung development, the involved regulatory elements should include both housekeeping and stage-specific ones.

It is well known that transcription factors (TFs) and microRNAs (miRNAs) are key regulators for gene expression regulation in high eukaryotes. The transcription factor play a key role in the transcription process which controls the activity of a gene by determining whether the gene's DNA is transcribed. The first step of the process of transcription is to convert the gene's DNA into a complementary coding mRNA molecule; then in the second step, the mRNA molecule is translated as an amino acid sequence of a protein [4]. While, miRNAs are a class of short RNA (21-24 nt) sequences that can regulate the expression of target genes at the post-transcriptional level [5]. Based on the pairing of miRNAs and their target sites on corresponding mRNAs, the complexes can inhibit translation by either degrading the mRNAs, or by blocking translation without degrading the targets [5].

There have been many studies focusing on investigating the functions of miRNAs or TFs, respectively. For examples, Dong *et al*. investigated the dynamical miRNA and mRNA regulation patterns for lung development by using statistical and computational methods [6]. Bandyopadhyay *et al*. obtained a global perspective of miRNA dysregulation in multiple cancer types by analyzing the differential expression patterns of specific miRNAs in cancer tissues [7]. There are also some studies considering the miRNA regulatory network [8-10] or TFs and target genes regulatory networks [11]. As a result, many computational tools have been proposed to predict the miRNA-mRNA regulations (EMBL [12], Miranda [13], SNMNMF[14,15], PicTar [16], mirConnX [17], and TargetScan [18]), or the TF-mRNA regulations (TRRD[19], JASPAR[20], and TRANSFAC [21,22]). Recently there are several attempts to discover the combinational links between miRNAs, TFs and target mRNAs (genes) [23-26], however, it is obviously that not enough attentions are paid on the regulatory combinations to understand the molecular mechanism of the complicated biological processes. In our preliminary version of this work [27], we have proposed a methodology to consider the regulatory combinations involved in the mouse lung development, and found some regulatory combinations in different lung developmental stages. Our earlier work has only considered the context of lung specific genes. However, the lung developmental process is complicated, which may be involved in not only lung specific but also lung non-specific genes and their regulators. Therefore, not just lung specific genes, but other tissue genes should also be considered to systematically investigate the regulatory mechanism of lung development.

It is widely acknowledged that cancer is a kind of complex developmental disorders, and some controlling developmental systems might also be involved in causing cancer. Lung cancer is the highest mortality cancer [28] and thus has recently attracted more and more attentions. Bonner et al. [29] have examined the genetic changes occurring during lung carcinogenesis by analyzing tissues from mice normal lungs and cancers on Affymetrix U74Av2 gene chips, and found that 24 developmentally regulated genes are aberrantly expressed in lung tumours. Kopantzev et al. [29,30] have found that opposite differences by comparing gene expression profiles in human non-small cell lung carcinomas and in human fetal lung development. Understanding the association of the regulatory mechanisms between lung development and lung tumour might provide novel insight of the lung carcinogenesis, which is helpful for the treatment of the lung cancer.

To address above questions, in this paper we define the significant triple relation among miRNA, TF and mRNA as a circuit, and propose to detect the circuits to identify the non-trivial regulatory combinations in the lung development of mouse. Then we analyze the combinations across the lung development and find out stage-specific combinations. According to the lung specific genes collected from the tissue specific gene database TiSGeD [31], we further distinguish circuits specific to lung development from those irrelevant to the lung developmental process. Finally, we consider the genes that have been shown to be related to cancers in literature and investigate their involved circuitsto find out the multiple roles of miRNAs and TFs in lung development and tumour progression. Our results give a systematic view for understanding the alteration of transcriptional or post-transcriptional regulatory factors and their roles involved in the mouse lung development and tumour progression.

## Methods

**Figure 1:**
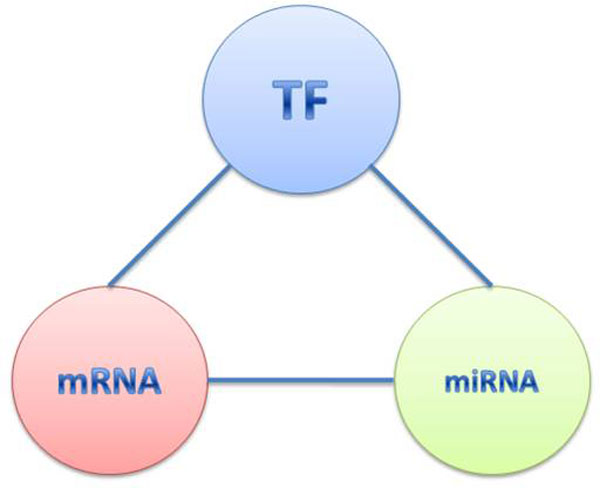
**The circuit structure**.

**Figure 2:**
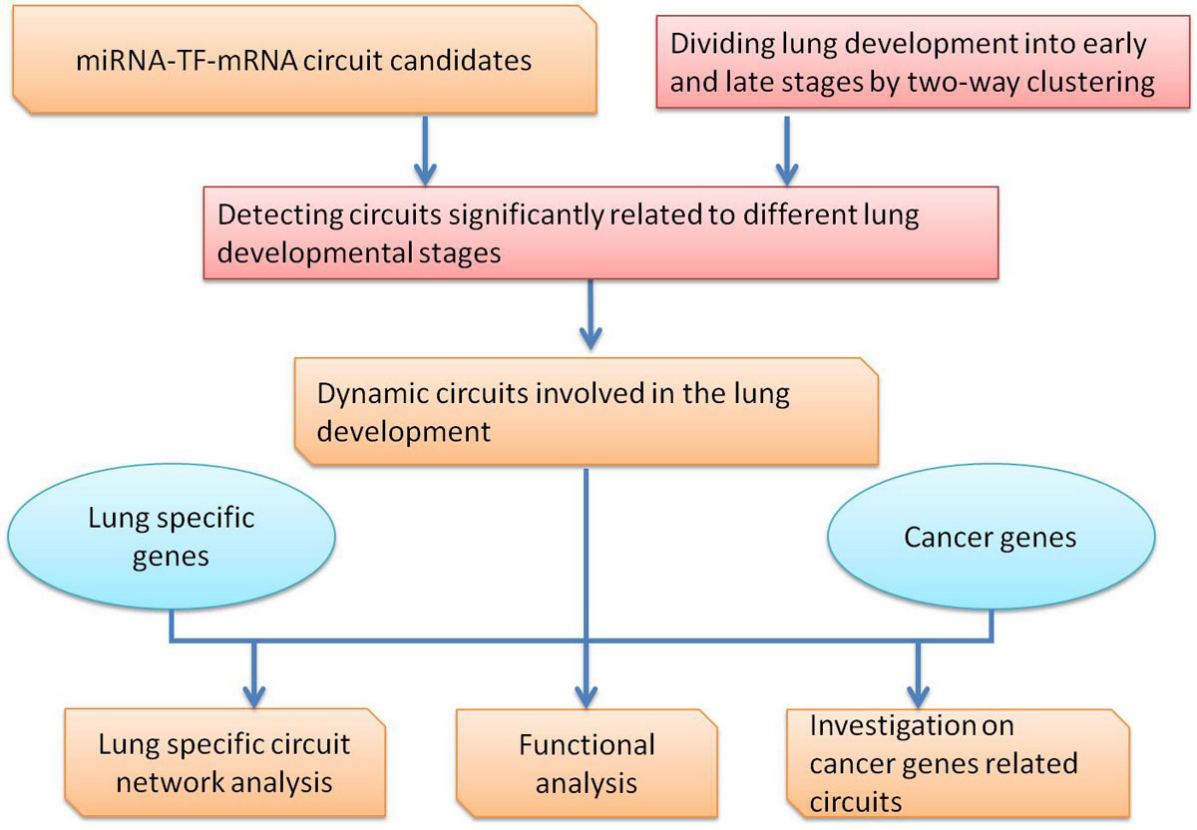
**Analysis procedure of circuits**.

In this paper, we define the triple relationship among mRNA, TF and miRNA as a circuit (Figure 1) in which the information from transcription and post-transcription is fused to impose and recognize non trivial regulatory combinations [32]. Based on the definition, we integrate information on miRNAs, TFs, genes, and their interactions to detect and investigate the circuits related to the biological process (Figure 2).

### Data resources

(1) Lung development time-course data collected from two mouse samples: GSE21053 [6]. GSE21053 consists of GSE20152 and GSE20954, corresponding to miRNA and gene expression data respectively. The expression values are measured at seven time points: embryo day 12, 14, 16, and 18; and postnatal day 2, 10 and 30.

(2) Lung specific genes: The Tissue-Specific Genes Database (TiSGeD) [31]. TiSGeD provides comprehensive information of genes specific to lung and other tissues collected from biomedical literatures. By setting SPM [31] threshold as 0.3, we extract 511 lung specific genes from TiSGeD, 455 of them are in common with GSE20954 and will be used for further study.

(3) Cancer related genes: we collect all the 487 cancer related genes from Cancer Gene Census[33], 118 of which are overlapped with the lung developmental genes, and 19 of which are also found to be lung specific genes.

(4) miRNA-gene pairs: miRanda [34], TargetScan [18] and circuitDB [35].

(5) TF-gene pairs: KEGG [36], Tred [37] and and circuitDB [35].

(6) miRNA-TF pairs: circuitDB [35].

### Extraction of miRNA-TF-mRNA circuit candidates

**Figure 3:**
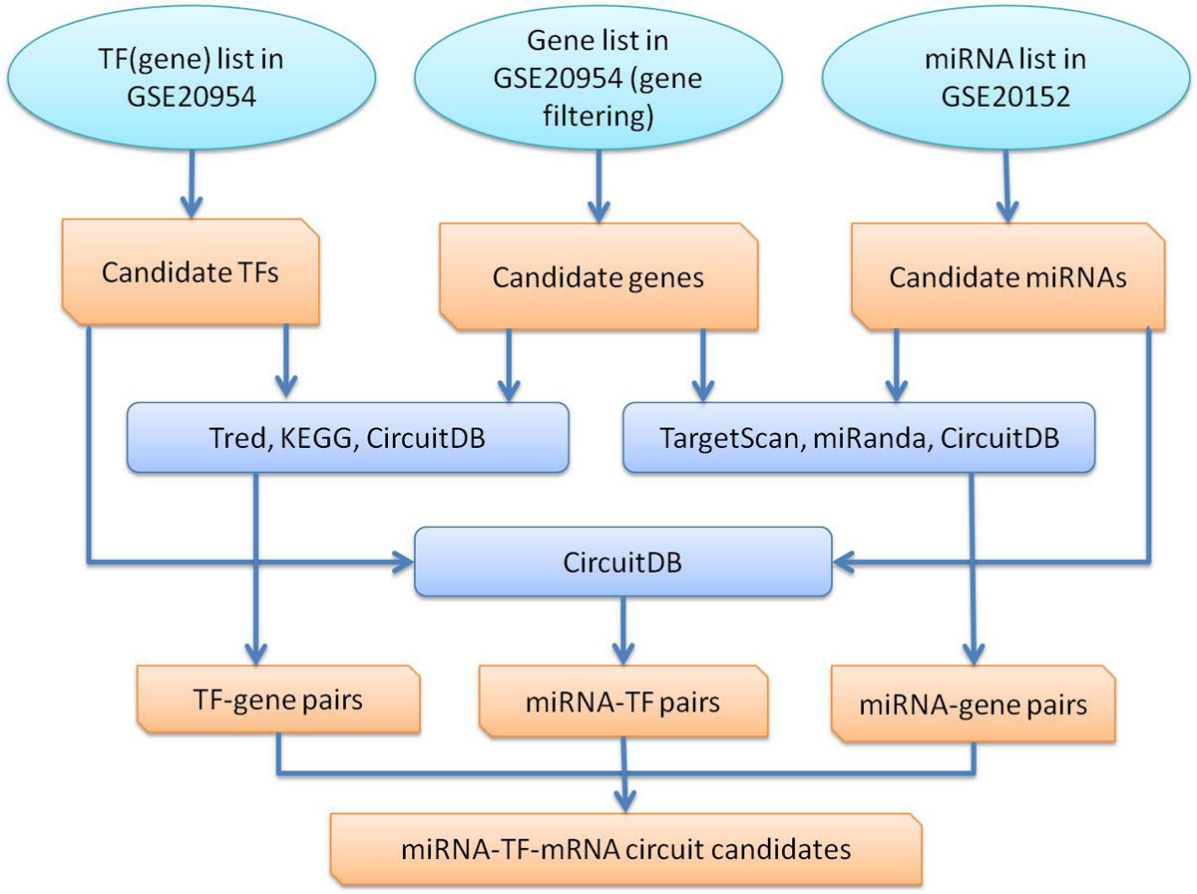
**Extraction of circuit candidates**.

The flowchart of extracting miRNA-TF-mRNA circuit candidates is shown in Figure 3. First, we extract miRNAs from GSE20152, TFs and genes from GSE20954 (we just use the coding genes to represent the TFs in this work). We preprocess gene expression data by filtering out those with small variances along all seven time points. As a result, there remain 8299 genes for further study. Then we extract miRNA-gene, TF-gene and miRNA-TF pairs from the data described above. All pairs are combined into potential miRNA-TF-mRNA circuits. As a result, we find 64760 circuit candidates, containing 8299 genes, 50 TFs and 118 miRNAs in total.

### Detection of significant circuits relevant to lung development

For each pair <*i*, *j*> in a circuit candidate (*i *and *j *are two vectors of expression data along a period of time points), we evaluate the significance of the pair-wise relationship at a specific stage of lung development by using Pearson correlation coefficient *R(i, j)*:

$$R\left(i,j\right)=\frac{\text{E}\left[{\left(i-\mu_{i}\right)}^{*}\left(j-\mu_{j}\right)\right]}{\sqrt{E\left[{\left(i-u_{i}\right)}^{*}\left(i-\mu_{i}\right)\right]E\left[{\left(j-\mu_{j}\right)}^{*}\left(j-\mu_{j}\right)\right]}}$$

where *E *is the mathematical expectation and *μ_i _*is the mean of vector *i*. We use the permutation test to evaluate the significance of the correlation and set the p-value threshold as 0.05. Only when all of three pairs in the same circuit candidate are significant, can it be regarded as a circuit at the specific period of lung development.

## Results and discussion

**Figure 4:**
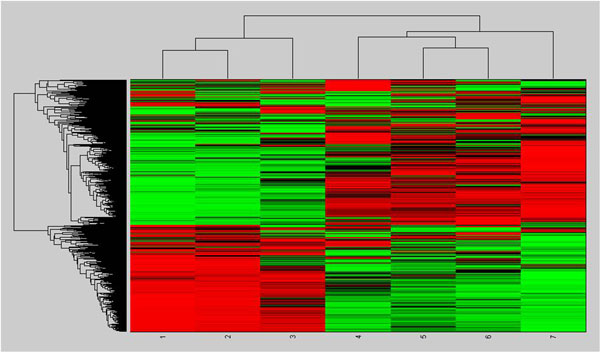
**Two-way clustering of genes**.

Figure 4 displays the result obtained by two-way clustering genes along 7 time points of the mouse lung development. It can be seen that the mouse lung development is mainly gone through two stages. The first stage is from time point 1 to time point 3 and the second stage is from time point 4 to time pint 7. We notice that although time points 1-4 correspond to embryo days 12, 14, 16 and 18, time point 4 is grouped with time points 5-7 (postnatal days 2, 10 and 30). Thus we can envision that the molecular mechanism of pseudoglandular and canalicular of lung (embryo days 12-17) is very different with that of saccular and alveolar (≥ embryo day 17) which continues until a period of time after birth (postnatal days 2, 10, and 30). Furthermore, we have found that time point 7 (postnatal day 30) are different with time points 4-6 though they are grouped together, which may be due to the fact that postnatal day 30 corresponds to the maturation at the microvasculature stage. Therefore, we only consider time points 1-3 and 4-6 of the lung development and respectively call them as early and late stages hereinafter.

**Figure 5:**
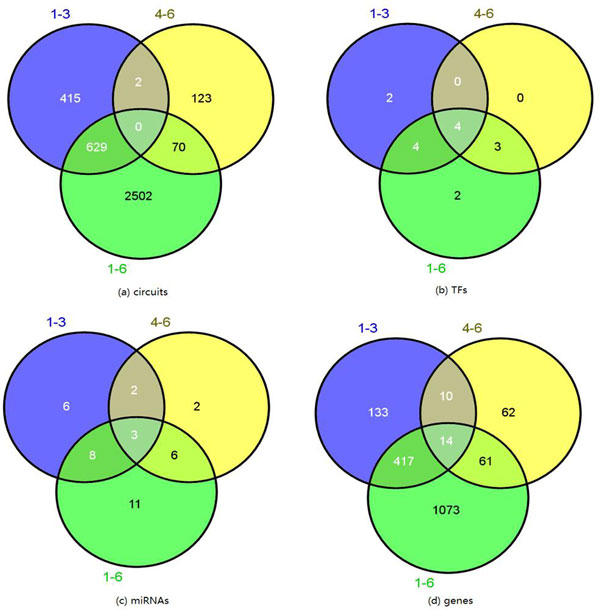
**Overlap in different lung development stages**. (a) Circuits overlap. (b) TF overlap. (c) miRNA overlap. (d) gene overlap.

From 64760 collected circuit candidates, we have mined 1046 circuits including 10 TFs, 574 genes and 19 miRNAs in the early stage, and 195 circuits including 7 TFs, 147 genes and 13 miRNAs in the late stage. To see how the circuits change across the lung development, we have also mined 3201 circuits involved in the whole six time points of the lung development, including 13 TFs, 1565 genes and 28 miRNAs. Figure 5 illustrates the overlap circuits among the early, late and whole stages. It can be seen that there are different circuits involved at different stages, which means that the roles of TFs/miRNAs/genes within the circuits vary greatly during the lung development, and they involve in different biological pathways at different stages.

### Dynamic circuits involved in the lung development

**Figure 6:**
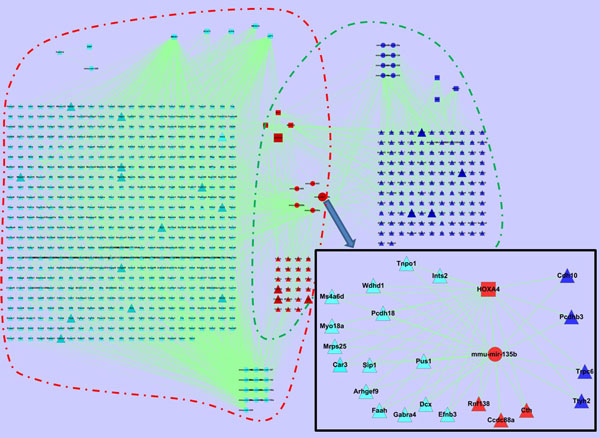
**Circuits in early and late stages of the lung development**. Circuits in red and green rectangles are of early and late stages respectively; and circle, square and triangle nodes stand for miRNAs, TFs and genes.

The circuits in the early and late stages of the lung development are shown in Figure 6 (see Additional file 1 for the details). We can see that different circuits involve in different stages. Even for those common miRNAs/TFs/genes, they participate in different circuits in two stages, showing that they may play varied roles during the lung development. For example, at the early stage the target genes of miRNA mir-135b are Arhgef9, Car3, Ccdc88a, Cth, Dcx, Efnb3, Faah, Gabra4, Ints2, Mrps25, Ms4a6d, Myo18a, Pcdh18, Pus1, Sip1, Tnpo1, and Wdhd1. However, at the late stage the target genes of miRNA mir-135b are changed to Ccdc88a, Cdh10, Cth, Pcdhb3, Rnf138, Trpc6, and Ttyh2.

**Figure 7:**
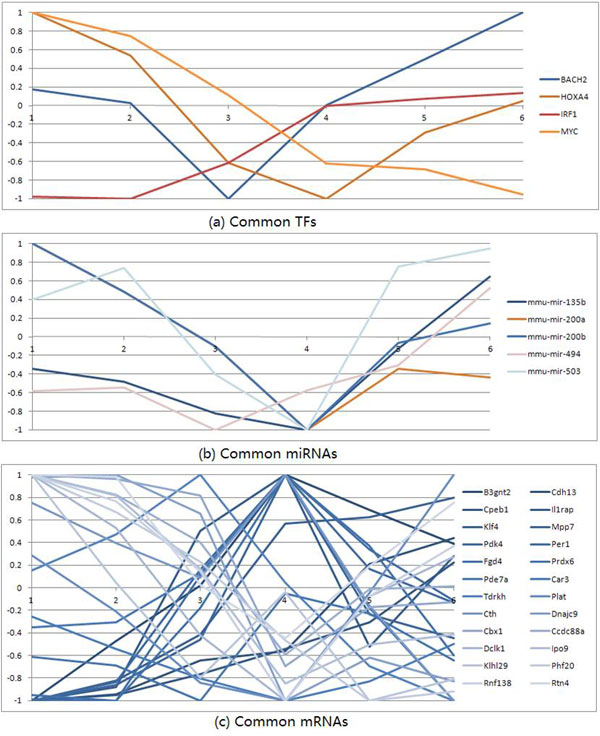
**Profiles of common TFs, miRNAs and genes at two stages of lung development**.

We further plot the expression profiles of common miRNAs/TFs/genes in Figure 7 to show the variations of their activities. From Figure 7 we can also see that the miRNAs/TFs/genes may be involved in different function modules in different stages.

#### TF overlap (Figure 5(b))

We have found that there are four TFs: BACH2, IRF1, MYC and HOXA4, which are participated in the early and late stages. BACH2 and HOXA4 are down-regulated in the early stage and up-regulated in the late stage (HOXA4 is one point time lagged); and IRF1 is up-regulated while MYC is down-regulated across all the time points (Figure 7(a)). It has been shown that Hox genes are implicated in the lung development and the patterning, and the HOXA4 and HOXA5 are likely to be involved in the patterning of the mouse lung [38]. According to the GO term GO:0048513, IRF1 and MYC also have the function related to the organ development. It is noticeable that MYC amplification is found to be a prognostic marker of early-stage lung adenocarcinoma [39], and BACH2 has also been researched in the cancer treatment [40], which shows that the tumour has association with the developmental process.

#### MiRNA overlap (Figure 5(c))

We have found that there are five miRNAs participated in both the early and late stages: mmu-mir-200a, mmu-mir-200b, mmu-mir-135b, mmu-mir-494 and mmu-mir-503. They show similar expression profiles along six time points of the lung development: first down-regulated and then up-regulated (Figure 7(b)). The miR-200 family is believed to play an essential role in the tumour suppression. It has been shown to play an important role in fibrotic lung diseases [41]. The mir-135b, mir-503b and mir-94 are also found to have relationship with lung cancer [42-44]. We think that miRNAs related to cancers may more likely participate in regulatory programs of both lung developmental stages.

#### Gene overlap (Figure 5(d))

We have found that there are 24 common genes roughly divided into two groups (Prdx6, B3gnt2, Car3, Cbx1, Ccdc88a, Cdh13, Cpeb1, Cth, Dclk1, Dnajc9, Fgd4, Il1rap, Ipo9, Klf4, Klhl29, Mpp7, Pde7a, Pdk4, Per1, Phf20, Plat, Rnf138, Rtn4, Tdrkh) occurring in the early and late stages (Figure 7(c)). It has been shown that most of them are related to tumours, such as Prdx6 [45], Cdh13 [46], Klf4 [47], and so on. Once again, we suggest that common genes in early and late stages of lung development may be more likely related to tumourigenesis even if they are not shown to be cancer genes.

### Dynamic regulations of circuits in the context of lung specific genes

**Figure 8:**
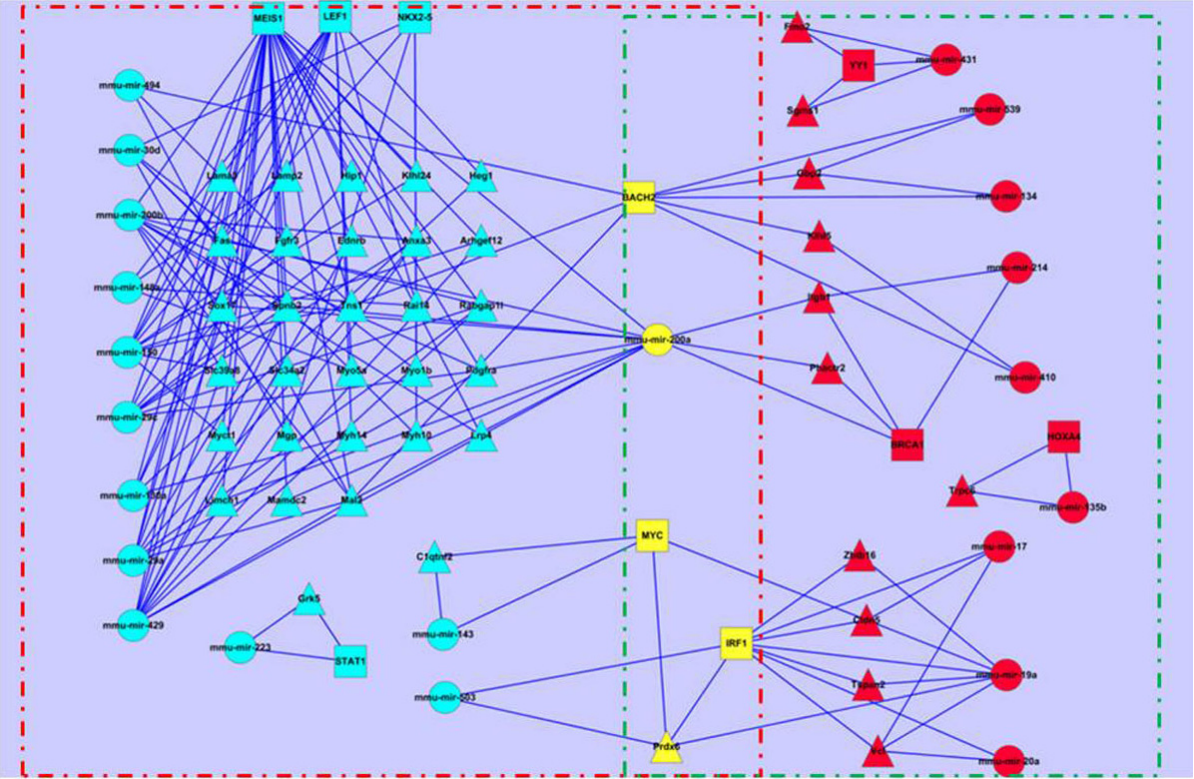
**The lung specific genes involved circuits at different stages**. Circuits in red and green rectangles are of early and late stages respectively; and circle, square and triangle nodes stand for miRNAs, TFs and genes respectively.

**Figure 9:**
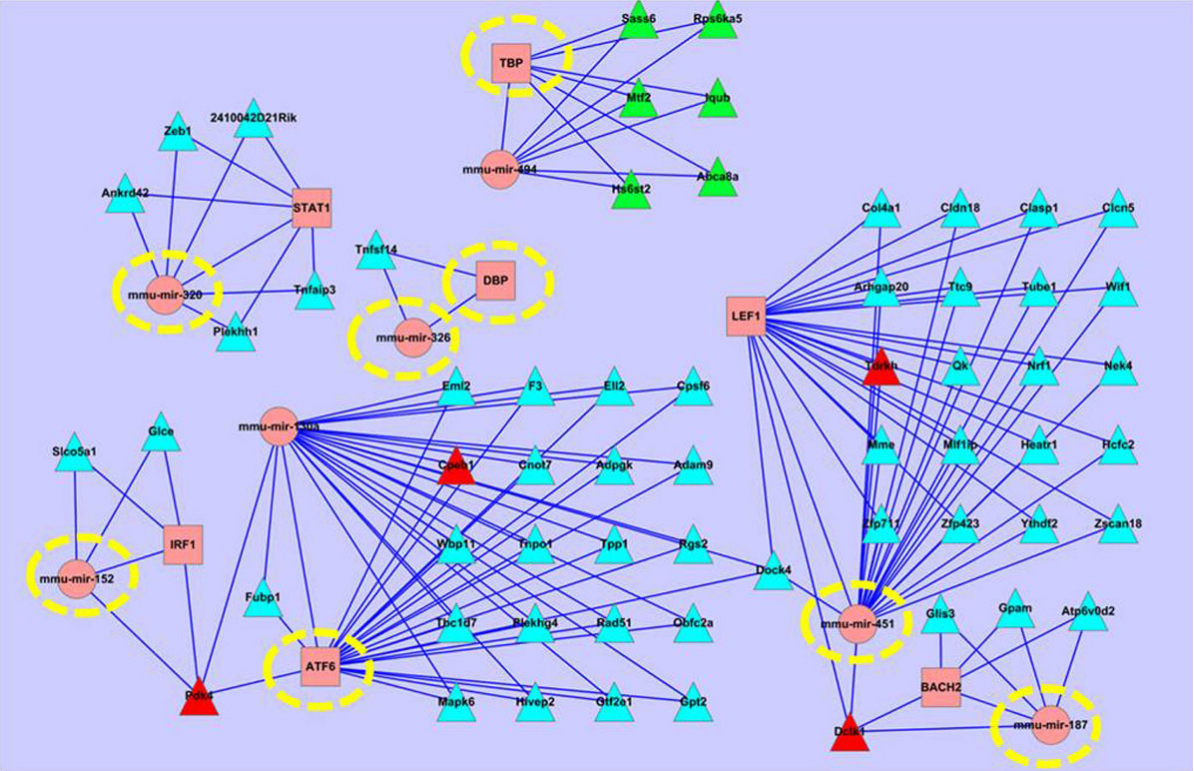
**Lung non-specific genes involved circuits**. TF and miRNA in dashed yellow circle box are in lung development circuit network but not are included in lung tissue specific circuit network.

Reinvestigating Figure 6 in the context of the lung specific genes, we have found that not only are lung specific but also non-specific genes related to the lung development (their involved circuits are shown in Figure 8 and Figure 9, respectively).

Specifically, we have detected out some regulators for lung non-specific genes, i.e., TF: ATF6, DBP (early), and TBP (late); miRNAs: mmu-mir-152, mmu-mir-187, mmu-mir-320, mmu-mir-326, mmu-mir-451 (early), and mmu-mir-494 (late). We think that they should have functions related to the lung development even though their regulating genes are not lung specific. For examples, ATF6 is an endoplasmic reticulum (ER) stress-regulated transmembrane transcription factor, and specific ER stress signaling transmitted by ATF6 leads to naturally occurring apoptosis during the muscle development [48]; DBP is a multifunctional protein found in plasma, ascitic fluid, cerebrospinal fluid, and urine and on the surface of many cell types; miR-320 plays a role in neuronal development[49]. According to the functional annotations of the regulators, it seems that they can actually play roles in the lung development in an indirect way.

**Figure 10:**
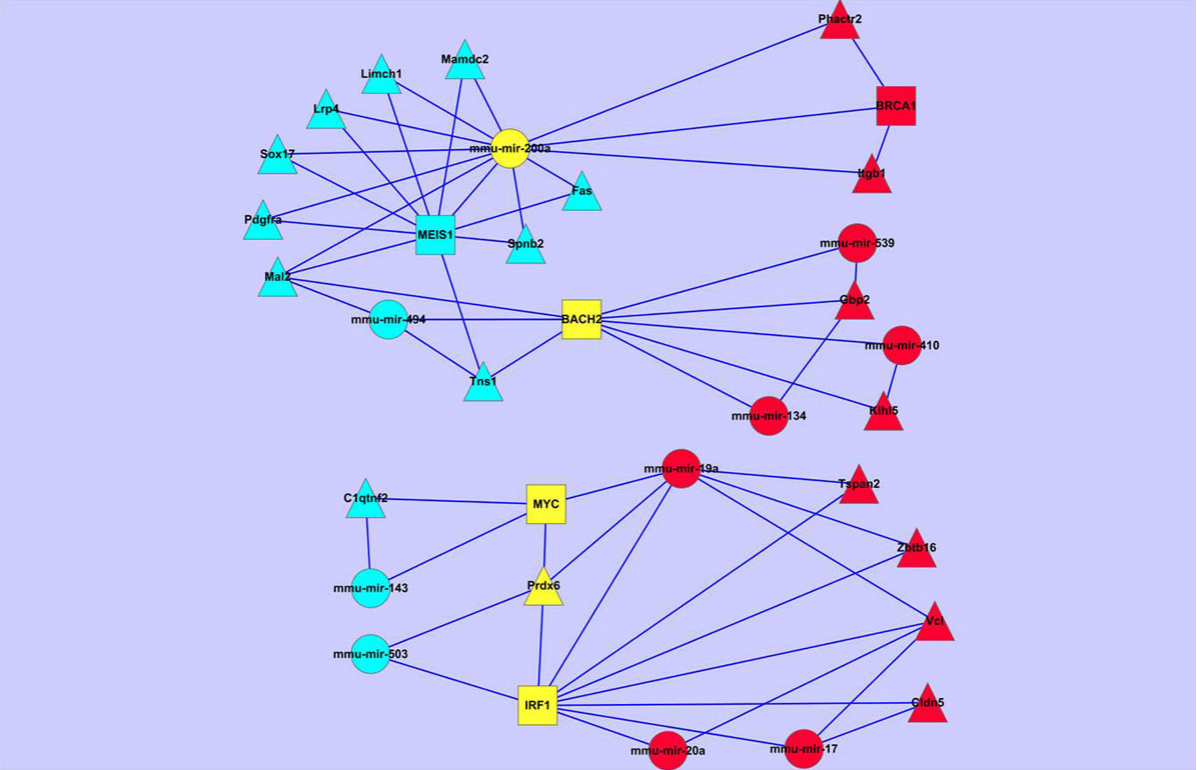
**The circuit network of common nodes at different stages**. Circuit network of the common TFs, miRNAs and gene in different lung development stages, in the context of lung specific genes.

To analyze the dynamic regulations in different stages of the lung development, we have constructed the circuit network of the common TFs, miRNAs and gene in different lung development stages, in the context of lung specific genes (as shown in Figure 10).

From Figure 10, we can see that different group of genes involved in early and late stages of the lung development, regulated by different TF-miRNA combinations. Moreover, some common genes/TFs/miRNAs involved in different stages show different functions. Gene Prdx6 is involved in one circuit in the early stage (IRF1~mmu-mir-503~Prdx6), while involved in the other two circuits containing the same miRNA (MYC~mmu-mir-19a~Prdx6, IRF1~mmu-mir-19a~Prdx6) in the late stage. Thus, in different stages of the lung development, Prdx6 may show different functions which are mainly determined by mmu-mir-503 and mmu-mir-19a, respectively.

We have also noted that mmu-mir-200a play some roles in the early stage by cooperating with MEIS1, while play other roles in the late stage by cooperating with BRCA1 together. By cooperating with different miRNAs, transcript factors BACH2, MYC or IF1 can co-regulate different genes in the early and late stages, respectively.

### Functional specificities of circuits in lung development

We have found that the miRNA and the TF usually have dynamical regulation programs at different stages during mouse lung development. In particular, the involved genes usually have opposite expression profiles at different stages (Figure 4). To computationally explore the potential functions of the dynamically regulated genes in lung development, we have employed biological process and pathway analysis on the lung specific genes.

Using DAVID tool [50], we get all enriched biological processes (Table 1) and pathways (Table 2) (P-value < 0.05). From Table 1, we have found several GO terms relevant to lung development. For example, three GO terms related to phosphorus function involve in the early stage: GO:0006793 GO:0006796 and GO:0016310. As known to all, inorganic phosphate (Pi) plays a critical role in diverse cellular functions and low Pi affects the lung development of mice by disturbing protein translation [51]. The GO:0051094 (positive regulation of developmental process) and GO:0035023 (regulation of Rho protein signal transduction) are also very relevant to the lung development. Rho protein plays a significant role in inhibiting lung development [52] while the ROCK2 plays a major role in the formation of the gas exchange units and as a sensor for accelerating the lung development [53,54].

**Table 1:** Top 10 GO biological process terms in different periods

Period	NO	Term	P-value
	1	**GO:0006793~phosphorus metabolic process**	5.40E-05
	2	**GO:0006796~phosphate metabolic process**	5.40E-05
	3	**GO:0016310~phosphorylation**	1.21E-04
	4	GO:0045596~negative regulation of cell differentiation	2.60E-04
**Early**	5	GO:0051056~regulation of small GTPase mediated signal transduction	3.32E-04
	6	GO:0006468~protein amino acid phosphorylation	5.28E-04
	7	**GO:0035023~regulation of Rho protein signal transduction**	5.30E-04
	8	GO:0007167~enzyme linked receptor protein signaling pathway	7.96E-04
	9	GO:0022604~regulation of cell morphogenesis	0.001564
	10	**GO:0051094~positive regulation of developmental process**	0.002006

	1	GO:0006928~cell motion	7.08E-05
	2	GO:0016477~cell migration	0.001009
	3	GO:0030030~cell projection organization	0.002595
	4	GO:0017148~negative regulation of translation	0.002614
**Late**	5	GO:0010558~negative regulation of macromolecule biosynthetic process	0.00269
	6	**GO:0010608~posttranscriptional regulation of gene expression**	0.002856
	7	GO:0032268~regulation of cellular protein metabolic process	0.003146
	8	GO:0031327~negative regulation of cellular biosynthetic process	0.003426
	9	GO:0048870~cell motility	0.003474
	10	GO:0051674~localization of cell	0.003474

**Table 2:** Significant pathways in different periods

Period	No	Term	P-value
	1	mmu04512:ECM-receptor interaction	0.005104
	**2**	**mmu04810:Regulation of actin cytoskeleton**	0.006057
**Early**	3	mmu04115:p53 signaling pathway	0.014693
	4	mmu05200:Pathways in cancer	0.028372
	**5**	**mmu00740:Riboflavin metabolism**	0.030496
	**6**	**mmu04060:Cytokine-cytokine receptor interaction**	0.036709

**Late**	**1**	**mmu00230:Purine metabolism**	0.011819
	**2**	**mmu00533:Keratan sulfate biosynthesis**	0.013904

In Table 2, the pathways indicated in bold have been reported to be associated with lung. For mmu04810, Actin cytoskeleton plays a role in human pulmonary artery ECS [55]. For mmu00740, lung remodelling induced by exposure of total parenteral nutrition to ambient light is due to the interaction between vitamin C and peroxides generated by the exposure of riboflavin to light [56]. For mmu04060, the analysis of lung adenocarcinoma tissue specimens have demonstrated that the genes involved in these biological pathways have high rates of over-expression [57]. For mmu00230, metabolic changes in the lung as a result of ventilation-induced lung injury are reflected by an increased level of purine in the bronchoalveolar lavage fluid and that purine may, thus, serve as an early marker for ventilation-induced lung injury [58]. For mmu00533, the pathways of Keratan sulphate biosynthesis are associated with prostate cancer and small cell lung cancer[59].

### Investigation on cancer genes involved in circuits

**Figure 11:**
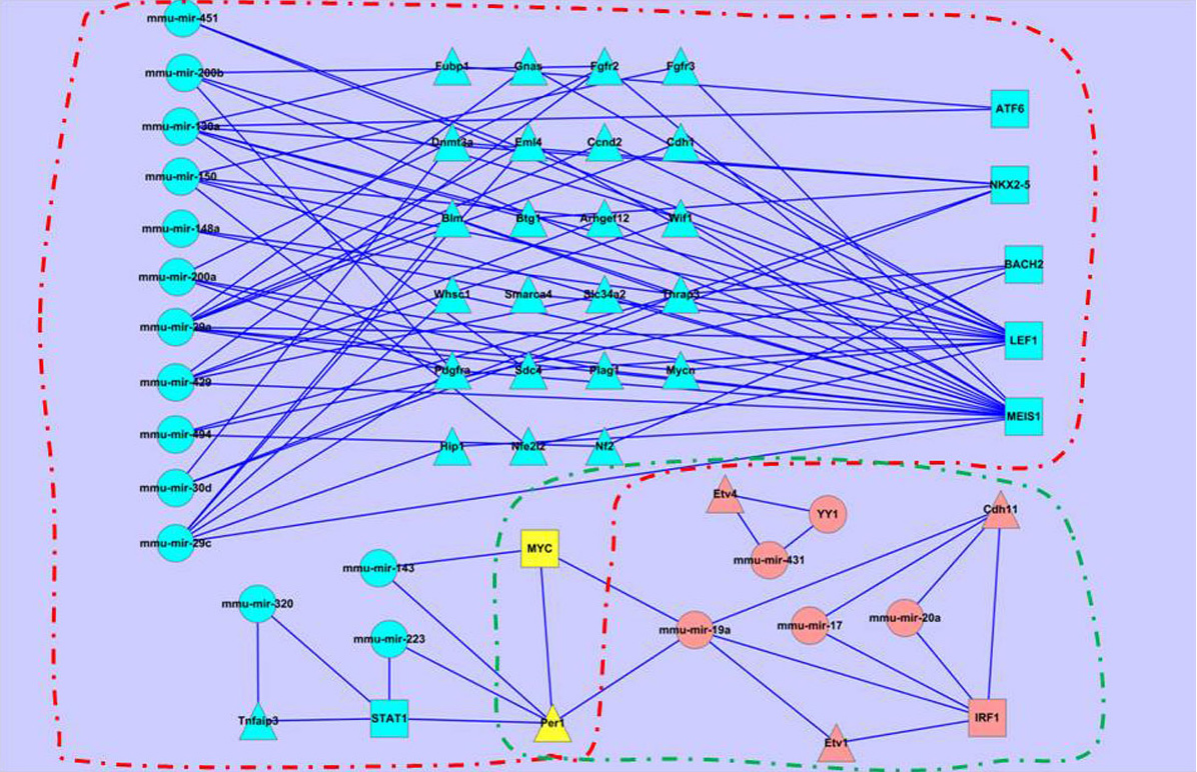
**Dynamic circuits related to cancer genes**. Circuits in red and green dashed box are of early and late stages respectively; and circle, square and triangle nodes stand for miRNAs, TFs and genes.

**Figure 12:**
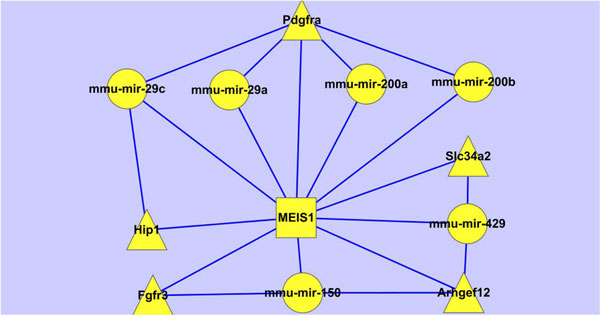
**Lung specific cancer genes involved circuits showing the association between lung development and tumourigenesis**.

As it is known to all, the developmentally regulated genes may be involved in lung tumours. Therefore we pick out the circuits related to the cancer genes from Figure 4 (shown in Figure 11). From Figure 11, we have identified out the regulating miRNAs and TFs of the 118 cancer genes and found out there are 38 circuits including 7 TFs, 14 miRNAs and 25 genes at the early stage; 6 circuits with 2 TFs, 4 miRNAs and 4 genes at the late stage. Any abnormal expression of the regulatory combinations may result in tumour. Compared to the cancer genes, the number of miRNAs/TFs is obviously smaller, which makes it more convenient to the research or treatment of cancer. From Figure 11, we have further extracted those lung specific genes involved circuits (shown in Figure 12), which contain 5 genes, 1 TFs and 6 miRNAs. We have noticed that miRNAs are from mir-200 family (mir-200a, mir-200b and mir-429), mir-29 family (mir-29a and mir-29c) and mir-150 family, they are all related to lung cancer[60,61]. Specifically, miR-200 miRNAs are involved in cancer metastasis[62]; mir-150 functions in hematopoiesis, and regulates genes whose downstream products encourage differentiating stem cells towards becoming megakaryocytes rather than erythrocytes [63]; mir-29 miRNAs activates p53, the tumour suppressor [64]. MEIS1 is a conserved transcription factor of the TALE-homeodomain class, expressed in a wide variety of tissues during development, and known to be required for the development of many organs in vertebrates and invertebrates. It has also been found incorrectly expressed in a number of tumour types, such as acute myeloid leukemia [65], lung adenocarcinoma tumours [66], nephroblastomas [67], and so on. Therefore, the circuits shown in Figure 12 uncovers that the regulatory combinations of MEIS1 and mir-200/mir-29/mir-150 miRNAs play roles in the early stage in the lung development, while their abnormal expressions may result in the tumour progression.

## Conclusions

In this work, we have mined and investigated the miRNA-TF-mRNA circuits involve in mouse lung development. The results have shown that the relevant transcriptional or post-transcriptional factors and their roles involved in lung development greatly vary at different stages. By considering those circuits associated with lung specific genes, we have identified out the dynamic regulatory interaction of miRNA-TF-mRNA circuits in different lung development stages. By investigating the circuits in the context of cancer genes, we have detected out some circuits related to the lung cancer, thus illustrating the association between the lung development and the tumourigenesis. Therefore, the miRNA-TF-mRNA circuits can be used in wide translational biomedicine studies, and can provide potential drug targets towards the treatment of lung cancer.

## Competing interests

The authors declare that they have no competing interests.

## Authors' contributions

Liu initiated the main idea of the paper and supervised the whole work. Ye conducted all of the programming and computational experiments. Liu, Ye and Wu discussed about this study. The manuscript was written by Liu and Ye, and revised by Liu, Ye and Wu.

## Supplementary Material

Additional file 1 **The excel file for the circuits shown in **Figure 6. Click here for file

## Declarations

The publication of this article has been funded by the National Science Foundation of China (61272274, 60970063), the program for New Century Excellent Talents in Universities (NCET-10-0644), the Ph.D. Programs Foundation of Ministry of Education of China (20090141110026) and the Fundamental Research Funds for the Central Universities (6081007).

This article has been published as part of *BMC Systems Biology *Volume 7 Supplement 2, 2013: Selected articles from The 6^th ^International Conference of Computational Biology. The full contents of the supplement are available online at http://www.biomedcentral.com/bmcsystbiol/supplements/7/S2.
